# A second polymorph of sodium di­hydrogen citrate, NaH_2_C_6_H_5_O_7_: structure solution from powder diffraction data and DFT comparison

**DOI:** 10.1107/S2056989016008343

**Published:** 2016-05-27

**Authors:** Alagappa Rammohan, James A. Kaduk

**Affiliations:** a Atlantic International University, Honolulu, HI, USA; b Illinois Institute of Technology, Chicago, IL, USA

**Keywords:** crystal structure, powder diffraction, density functional theory, citrate, sodium, polymorphs

## Abstract

The crystal structure of a second polymorph of sodium di­hydrogen citrate, Na^+^·H_2_C_6_H_5_O_7_^−^, has been solved and refined using laboratory X-ray powder diffraction data, and optimized using density functional techniques. The powder pattern of the commercial sample used in this study did not match that corresponding to the known crystal structure [Glusker *et al.* (1965). *Acta Cryst*. **19**, 561–572; refcode NAHCIT]. In this polymorph, the [NaO_7_] coordination polyhedra form edge-sharing chains propagating along the *a* axis, while in NAHCIT the octa­hedral [NaO_6_] groups form edge-sharing pairs bridged by two hy­droxy groups. The most notable difference is that in this polymorph one of the terminal carboxyl groups is deprotonated, while in NAHCIT the central carboxyl­ate group is deprotonated, as is more typical.

## Abstract 2

The crystal structure of a second polymorph of sodium di­hydrogen citrate has been solved and refined using laboratory X-ray powder diffraction data, and optimized using density functional techniques. The powder pattern of a commercial sample did not match that corresponding to the known crystal structure (NAHCIT).

## Chemical context   

In the course of a systematic study of the crystal structures of Group 1 (alkali metal) citrate salts to better understand the anion’s conformational flexibility, deprotonation mode, coordination tendencies, and hydrogen bonding, we have determined several new crystal structures. Most of the new structures were solved using powder diffraction data (laboratory and/or synchrotron), but single crystals were used where available. The general trends and conclusions about the 16 new compounds and 12 previously characterized structures are being reported separately (Rammohan & Kaduk, 2016*a* ▸). Three of the new structures – NaKHC_6_H_5_O_7_, NaK_2_C_6_H_5_O_7_, and Na_3_C_6_H_5_O_7_ – have been published recently (Rammohan & Kaduk, 2016*b* ▸,*c* ▸,*d* ▸).
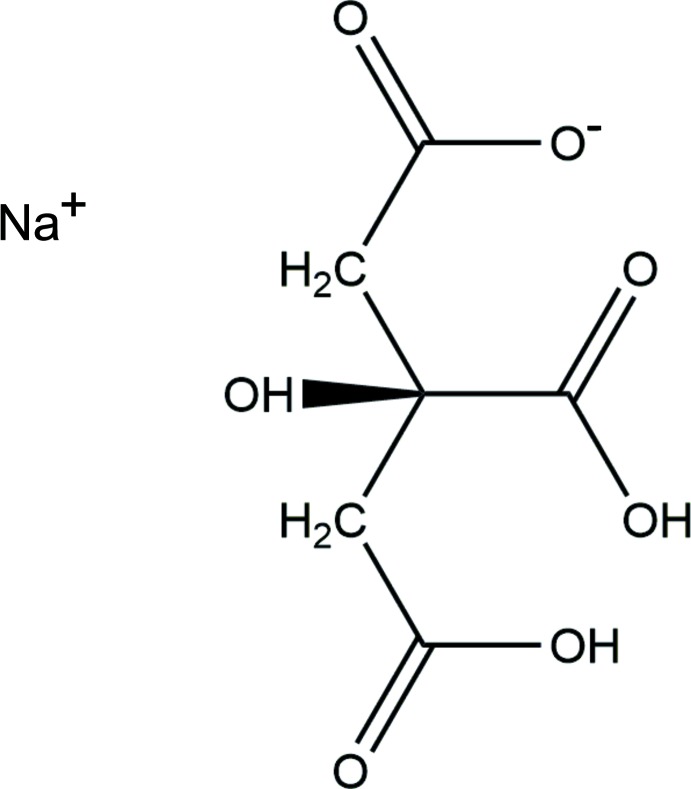


## Structural commentary   

**Figure 1:**
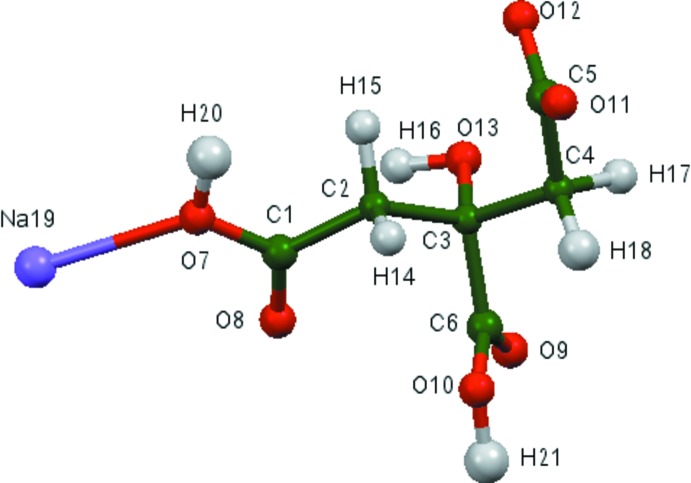
The asymmetric unit, showing the atom numbering. The atoms are represented by 50% probability spheroids.

**Figure 2:**
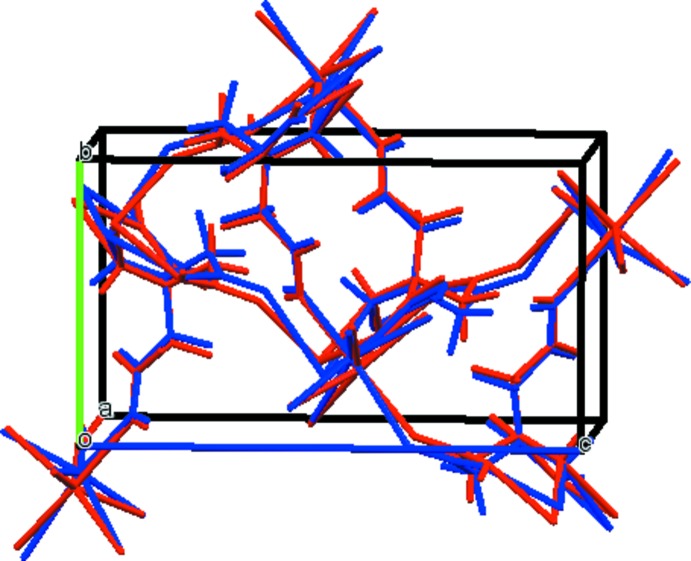
Comparison of the refined and optimized structures of sodium di­hydrogen citrate. The refined structure is in red, and the DFT-optimized structure is in blue.

The asymmetric unit of the title compound is shown in Fig. 1 ▸. The root-mean-square deviation of the non-hydrogen atoms in the Rietveld-refined and DFT-optimized structures is 0.148 Å. The maximum deviation is 0.318 Å, at the sodium ion. The good agreement between the two structures (Fig. 2 ▸) is strong evidence that the experimental structure is correct (van de Streek & Neumann, 2014 ▸). This discussion uses the DFT-optimized structure. All of the bond lengths, bond angles, and most torsion angles fall within the normal ranges indicated by a *Mercury Mogul* geometry check (Macrae *et al.*, 2008 ▸). Only the C2—C3—C4—C5 torsion angle is flagged as unusual. It lies in the tail of a minority *gauche* population of similar torsion angles. The citrate anion occurs in the *gauche,trans*-conformation, which is one of the two low-energy conformations of an isolated citrate ion. The central carboxyl­ate group and the hy­droxy group occur in the normal planar arrangement. The citrate chelates to one Na19 ion through the central carboxyl O9 atom and the hy­droxy group O13, and to a second Na19 ion through the terminal carboxyl atom O12 and the hy­droxy group O13. The Na^+^ ion is seven-coordinate (penta­gonal–bipyramidal), and has a bond-valence sum of 1.12.

## Supra­molecular features   

**Figure 3:**
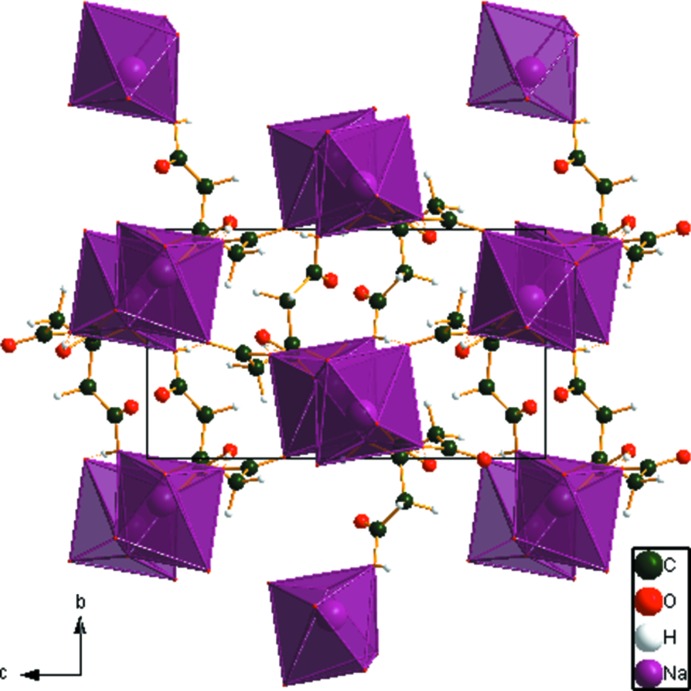
Crystal structure of NaH_2_C_6_H_5_O_7_, viewed down the *a* axis.

In this polymorph, the [NaO_7_] coordination polyhedra (Fig. 3 ▸) form edge-sharing chains propagating along the *a* axis, while in NAHCIT (Glusker *et al.*, 1965 ▸), the octa­hedral [NaO_6_] units form edge-sharing pairs bridged by two hy­droxy groups.

The conformations of the citrate ions in the two structures are similar. The root-mean-square displacement of the non-hydrogen atoms is 0.11 Å. The conformations of the hy­droxy groups differ, reflecting differences in coordination and hydrogen bonding. The most notable difference is that in this polymorph, one of the terminal carboxyl groups is deprotonated, while in NAHCIT the central carboxyl­ate group is deprotonated, as is more typical.

In this form, the hydrogen bonds occur in layers in the *ab* plane, while in NAHCIT the hydrogen bonds form double-ladder chains along the *c* axis. The hydrogen bonds in this form contribute about 4.3 kcal mol^−1^ more to the lattice energy than those in NAHCIT, and seem to include a C—H⋯O hydrogen bond (Table 1 ▸). Comparison of the DFT energies of the two polymorphs shows that this polymorph is 3.24 kcal mol^−1^ higher in energy than NAHCIT. Presumably it was crystallized at a higher temperature than NAHCIT, which was crystallized at 343 K.

## Database survey   

Details of the comprehensive literature search for citrate structures are presented in Rammohan & Kaduk (2016*a* ▸). The crystal structure of sodium di­hydrogen citrate is reported in Glusker *et al.* (1965 ▸), and the powder pattern calculated from this structure is PDF entry 02-063-5032. The observed powder pattern matched PDF entry 00-016-1182 (de Wolff *et al.*, 1966 ▸) A reduced cell search of the cell of the observed polymorph in the Cambridge Structural Database (Groom *et al.*, 2016 ▸) (increasing the default tolerance from 1.5 to 2.0%) yielded 223 hits, but limiting the chemistry to C, H, Na, and O only resulted in no hits. The powder pattern is now contained in the the Powder Diffraction File (ICDD, 2015 ▸) as entry 00-063-1340.

## Synthesis and crystallization   

The sample was purchased from Sigma–Aldrich (lot #BCBC0142). Before measuring the powder pattern, a portion of the sample was ground in a Spex 8000 mixer/mill and blended with a NIST SRM 640b silicon inter­nal standard.

## Refinement details   

The powder pattern was indexed using DICVOL06 (Louër & Boultif, 2007 ▸). The background and *K*α_2_ peaks were removed using *Jade* (MDI, 2012 ▸), and *Powder4* (Dragoe, 2001 ▸) was used to convert the data into an XYE file. The 10–52.22° portion of the pattern was processed in *DASH 3.2* (David *et al.*, 2006 ▸), which suggested *P*2_1_2_1_2_1_ as the most probable space group. Citrate and Na fragments were used to solve the structure in this space group using *DASH*.

**Figure 4:**
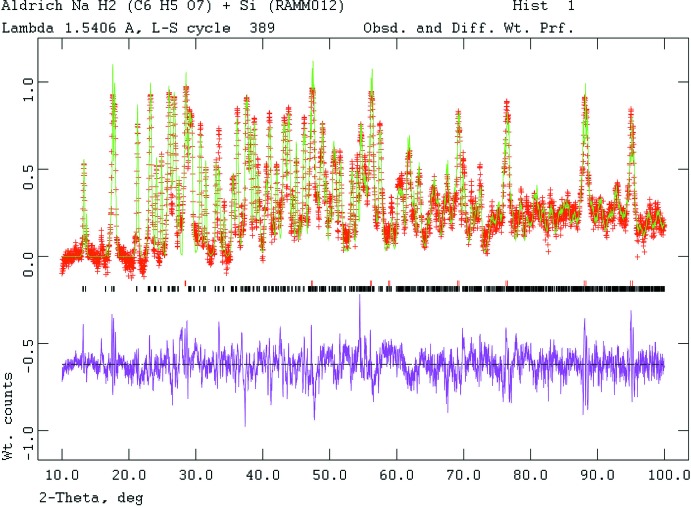
Rietveld plot for the refinement of NaH_2_C_6_H_5_O_7_. The vertical scale is not the raw counts but the counts multiplied by the least-squares weights. This plot emphasizes the fit of the weaker peaks. The red crosses represent the observed data points, and the green line is the calculated pattern. The magenta curve is the difference pattern, plotted at the same scale as the other patterns. The lower row of black tick marks indicates the reflection positions for the major phase and the upper row of red tick marks is for the silicon inter­nal standard.

The powder pattern (Fig. 4 ▸) was indexed using *Jade 9.5* (MDI, 2012 ▸). Pseudo-Voigt profile coefficients were as parameterized in Thompson *et al.* (1987 ▸) with profile coefficients for Simpson’s rule integration of the Pseudo-Voigt function according to Howard (1982 ▸). The asymmetry correction of Finger *et al.* (1994 ▸) was applied and microstrain broadening by Stephens (1999 ▸).

The structure was refined by the Rietveld method using *GSAS/EXPGUI* (Larson & Von Dreele, 2004 ▸: Toby, 2001 ▸). All C—C and C—O bond lengths were restrained, as were all bond angles. The hydrogen atoms were included at fixed positions, which were recalculated during the course of the refinement using *Materials Studio* (Dassault Systemes, 2014 ▸). The *U*_iso_ values of the atoms in the central and outer portions of the citrate were constrained to be equal, and the *U*_iso_ values of the hydrogen atoms were constrained to be 1.3× those of the atoms to which they are attached.

The Bravais–Friedel–Donnay–Harker (Bravais, 1866 ▸; Friedel, 1907 ▸; Donnay & Harker, 1937 ▸) morphology suggests that we might expect a blocky morphology for this phase. A 4th-order spherical harmonic texture model was included in the refinement. The texture index was 1.374, indicating that preferred orientation was significant for this rotated-flat-plate specimen.

## DFT calculations   

Crystal data, data collection and structure refinement details are summarized in Table 2 ▸. After the Rietveld refinement, a density functional geometry optimization (fixed experimental unit cell) was carried out using *CRYSTAL09* (Dovesi *et al.*, 2005 ▸). The basis sets for the C, H, and O atoms were those of Gatti *et al.* (1994 ▸), and the basis set for Na was that of Dovesi *et al.* (1991 ▸). The calculation used 8 *k*-points and the B3LYP functional, and took about 60 h on a 2.4 GHz PC. The *U*_iso_ from the Rietveld were assigned to the optimized fractional coordinates.

## Supplementary Material

Crystal structure: contains datablock(s) RAMM012A_publ, ramm012a_DFT, RAMM012A_overall, RAMM012A_phase_1, RAMM012A_phase_2, RAMM012A_p_01. DOI: 10.1107/S2056989016008343/hb7585sup1.cif

CCDC references: 1481347, 1481346, 1481345

Additional supporting information:  crystallographic information; 3D view; checkCIF report

## Figures and Tables

**Table 1:** Hydrogen-bond geometry (Å, °)

*D*—H⋯*A*	*D*—H	H⋯*A*	*D*⋯*A*	*D*—H⋯*A*
O7—H20⋯O11	1.01	1.61	2.627	176
O10—H21⋯O12	1.04	1.46	2.498	175
O13—H16⋯O8	0.97	2.50	3.033	114
C2—H15⋯O8	1.09	2.50	3.166	119

**Table 2:** Experimental details

	Phase 1	Phase 2
Crystal data		
Chemical formula	Na^+^·C_6_H_7_O_7_^−^	Si
*M*_r_	214.10	28.09
Crystal system, space group	Orthorhombic, *P*2_1_2_1_2_1_	Cubic, *F* *d* 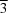 *m*
Temperature (K)	300	300
*a*, *b*, *c* (Å)	7.4527 (3), 7.7032 (3), 13.4551 (4)	5.43105, 5.43105, 5.43105
α, β, γ (°)	90, 90, 90	90, 90, 90
*V* (Å^3^)	772.45 (5)	160.20
*Z*	4	8
Radiation type	*K*α_1_, *K*α_2_, λ = 1.540629, 1.544451 Å	*K*α_1_, *K*α_2_, λ = 1.540629, 1.544451 Å
Specimen shape, size (mm)	Flat sheet, 25 × 25	Flat sheet, 25 × 25

Data collection		
Diffractometer	Bruker D2 Phaser	Bruker D2 Phaser
Specimen mounting	Bruker PMMA holder	Bruker PMMA holder
Data collection mode	Reflection	Reflection
Scan method	Step	Step
2θ values (°)	2θ_min_ = 5.042 2θ_max_ = 100.048 2θ_step_ = 0.020	2θ_min_ = 5.042 2θ_max_ = 100.048 2θ_step_ = 0.020

Refinement		
*R* factors and goodness of fit	*R*_p_ = 0.063, *R*_wp_ = 0.084, *R*_exp_ = 0.024, *R*(*F* ^2^) = 0.0780, χ^2^ = 12.180	*R*_p_ = 0.063, *R*_wp_ = 0.084, *R*_exp_ = 0.024, *R*(*F* ^2^) = 0.0780, χ^2^ = 12.180
No. of parameters	76	76
No. of restraints	29	29

The same symmetry and lattice parameters were used for the DFT calculation. Computer programs: *DIFFRAC.Measurement* (Bruker, 2009 ▸), *Powder4* (Dragoe, 2001 ▸), *DASH* (David *et al.*, 2006 ▸), *GSAS* (Larson & Von Dreele, 2004 ▸), *EXPGUI* (Toby, 2001 ▸), *DIAMOND* (Crystal Impact, 2015 ▸) and *publCIF* (Westrip, 2010 ▸).

## supplementary crystallographic information

### Crystal data

**Table:**

C_6_H_7_NaO_7_	*c* = 13.4551 Å
*M**_r_* = 214.10	*V* = 772.45 Å^3^
Orthorhombic, *P*2_1_2_1_2_1_	*Z* = 4
*a* = 7.4527 Å	None; DFT calculation radiation
*b* = 7.7032 Å	*T* = 300 K

### Fractional atomic coordinates and isotropic or equivalent isotropic displacement parameters (Å^2^)

**Table:**

	*x*	*y*	*z*	*U*_iso_*/*U*_eq_	
C1	0.86930	0.18689	0.07976	0.02580*	
C2	0.76864	0.31082	0.14718	0.01910*	
C3	0.80887	0.50489	0.13526	0.01910*	
C4	0.72870	0.60356	0.22535	0.01910*	
C5	0.54276	0.54256	0.25725	0.02580*	
C6	1.01121	0.54559	0.13579	0.02580*	
O7	0.79690	0.02935	0.07081	0.02580*	
O8	1.01065	0.22287	0.03877	0.02580*	
O9	1.07983	0.64886	0.07729	0.02580*	
O10	1.09698	0.46975	0.20891	0.02580*	
O11	0.51997	0.49361	0.34617	0.02580*	
O12	0.42047	0.54898	0.19138	0.02580*	
O13	0.73153	0.57264	0.04623	0.02580*	
H14	0.79934	0.27249	0.22351	0.02500*	
H15	0.62504	0.29365	0.13548	0.02500*	
H16	0.75707	0.49090	−0.00712	0.02500*	
H17	0.71973	0.74075	0.20546	0.02500*	
H18	0.81890	0.59174	0.28862	0.03350*	
Na19	0.91363	−0.18930	−0.03840	0.03460*	
H20	0.67516	0.02054	0.10415	0.03900*	
H21	0.23334	0.49718	0.20429	0.03900*	

### Bond lengths (Å)

**Table:**

C1—C2	1.516	C4—H17	1.092
C1—O7	1.334	C4—H18	1.089
C1—O8	1.221	C5—O11	1.266
C2—C3	1.533	C5—O12	1.272
C2—H14	1.093	C6—O9	1.230
C2—H15	1.090	C6—O10	1.311
C3—C4	1.550	O7—H20	1.014
C3—C6	1.540	O10—H21^i^	1.040
C3—O13	1.428	O13—H16	0.974
C4—C5	1.525	H21—O10^ii^	1.040

Symmetry codes: (i) *x*+1, *y*, *z*; (ii) *x*−1, *y*, *z*.

### Hydrogen-bond geometry (Å, º)

**Table:**

*D*—H···*A*	*D*—H	H···*A*	*D*···*A*	*D*—H···*A*
O7—H20···O11	1.01	1.61	2.627	176
O10—H21···O12	1.04	1.46	2.498	175
O13—H16···O8	0.97	2.50	3.033	114
C2—H15···O8	1.09	2.50	3.166	119
